# Supplementary material for: Adverse birth outcomes and associated factors among newborns delivered in Sao Tome & Principe: A case‒control study
Source: PLoS One. 2023 Jul 7;18(7):e0276348. doi: 10.1371/journal.pone.0276348 (PMC10328319; doi:10.1371/journal.pone.0276348)
Supplement: Attachment — Submitted filename: Reply Editorial Manager.docx [file pone.0276348.s002.docx]

**PONE-D-22-27043**
Adverse birth outcomes and associated factors among newborns delivered in a western African country: a case‒control study

21 October, 2022

Dear Juvelyn A. Moreno,

Greetings

Question:

1) Thank you for submitting your documents.

Upon assessing the translated version, it does suggest that ethical approval has been received; however, we need to receive a copy of the ethical authorization that was obtained from the Ministry of Health of the Democratic Republic of São Tomé and Príncipe.

Once received we will be able to proceed.

Author´s reply;

At the time of the study protocol was submitted (year 2016), there were no ethics committees in STP; therefore, there was no study approval number for our study.

Nonetheless, the Ministry of Health of Sao Tome & Principe and the main board of the Hospital Dr. Ayres de Menezes are dedicated ethics oversight bodies, and both approved this study.

We have already submitted the study approval document (original document plus translation to EN).

I understand your request although the only documented that I received was from the main board of Hospital Dr Ayres de Menezes (HAM).

The Ministry of Health of the Democratic Republic of Sao Tome & Principe received our study protocol months before and approved it using the main board of HAM to communicate it to us, since the Ministry of Health would not communicate directly with the researcher.

Thus, the main board of HAM informed us that *“Clinical study had formal ethical authorization from the Ministry of Health of the Democratic Republic of São Tomé and Príncipe.” (see ethics approval document)*

Moreover, all methods in our study were performed in accordance with the relevant guidelines and regulations in practice.

Written informed consent was obtained from all participants (or their parent or legal guardian in the case of adolescent under 16 or for illiterate pregnant women) after the purpose of the research was explained orally by the principal investigator.

Participants' or their legal representatives' also consented to have the results of this research work published.

Participation in the survey was voluntary, as participants could decline to participate at any time during the study.

It is important to take into special consideration that this country is a low-Income country, the second smallest in Africa and a very resource-constrained with few research activities. There are no more than 10 published articles from the clinical area conducted in STP.

Nonetheless, our study was planned and conducted by the Portuguese Instituto de Higiene e Medicina Tropical – Global Health and Tropical Medicine from Universidade Nova de Lisboa – Portugal.

It was submitted to a grant that was given by the Fundação para a Ciência e Tecnologia (FCT).

So, this could only happen with the verification that all steps were taken to comply with all ethics requirements.

Please do take into consideration that in this study we only asked for reviewing the antenatal pregnancy card and abstract information from medical records along with an interview related to sociodemographic data from the newborn´s mother, father and family.

We now propose the following information to be added in the following section:

“Ethics approval and consent to participate

The study complies with the Declaration of Helsinki and was approved and consented to by the Ministry of Health of Sao Tome & Principe and by the main board of Hospital Dr. Ayres de Menezes, since at the time the study protocol was submitted there was no ethics committee in STP. Only recently has the country National Ethics Committee been appointed. Previously, study analysis and approval were performed by dedicated ethics oversight bodies, such as the Ministry of Health of Sao Tome & Principe and the main board of the Hospital Dr. Ayres de Menezes, and both approved this study.

Moreover, all methods in our study were performed in accordance with the relevant guidelines and regulations in practice.

Written informed consent was obtained from all participants after the purpose of the research was explained orally by the researcher. Approval by the participants´ parents or legal guardians was asked in the case of adolescents under 16 years of age or for illiterate women.

The participants or their legal representatives also consented to have the results of this research work to be published.

Participation in the survey was voluntary, as participants could decline to participate at any time during the study.”

2) Please could you also ensure that you provide copies of any related manuscripts as 'Other' files, for instance PONE-D-22-21368, as outlined in our submission guidelines which states that if related work has been submitted to PLOS ONE or elsewhere, authors must include a copy with the submitted article. Reviewers will be asked to comment on the overlap between related submissions.

Authors´s reply: Done.

We hope you find our manuscript suitable for publication and look forward to hearing from you in due course.

Respectfully,

Alexandra Vasconcelos,

Paediatrician

Unidade de Clínica Tropical

Global Health and Tropical Medicine (GHTM)_Instituto de Higiene e Medicina Tropical (IHMT), Universidade Nova de Lisboa (UNL)

https://www.ihmt.unl.pt/

Rua da Junqueira Nº100, 1349-008 Lisboa -Portugal

Telephone: +351 213 652 600 _ Email: alexandravasc@gmail.com
