# Supplementary material for: Adverse birth outcomes and associated factors among newborns delivered in Sao Tome & Principe: A case‒control study
Source: PLoS One. 2023 Jul 7;18(7):e0276348. doi: 10.1371/journal.pone.0276348 (PMC10328319; doi:10.1371/journal.pone.0276348)
Supplement: Attachment — Submitted filename: Response to Reviewers PLOS_PONE-D-22-27043.docx [file pone.0276348.s004.docx]

Rebuttal letter

Point-by-point response to Reviewers

Dear Editor and Reviewers,

On behalf of all authors, we express our gratitude to you for the constructive review of our manuscript PONE-D-22-27043

Adverse birth outcomes and associated factors among newborns delivered in a western African country: a case‒control study

PLOS ONE

We truly appreciate the Academic Editor Calistus Wilunda DrPH and Reviewer´s Lydia Sandrah Kaforau and Dr Amanuel Abajobir feedback and precious contributions.

We now submit a revised version of the manuscript that addresses the points raised during the review process.

In the following detailed response, we address each comment calling for changes point-by-point, indicating where relevant additional texts have been added to the body of the manuscript.

We submit a manuscript with track changes (file labeled 'Revised Manuscript with Track Changes') and a clean version of the review manuscript (“Manuscript”).

We believe that with the changes made, the manuscript has improved substantively in view of being considered for publication.

___________________________________________________

Journal Requirements:

1. Please ensure that your manuscript meets PLOS ONE's style requirements, including those for file naming. The PLOS ONE style templates can be found at

<https://journals.plos.org/plosone/s/file?id=wjVg/PLOSOne_formatting_sample_main_body.pdf>

and <https://journals.plos.org/plosone/s/file?id=ba62/PLOSOne_formatting_sample_title_authors_affiliations.pdf>

Authors’ response:

Our manuscript now meets PLOS ONE's style requirements.

Additional Editor Comments:

Please clarify the exposure variables. Some of the variables are mediators and it is unclear why they have been included in the analysis. The model building strategy is not clear.

Some other important risk factors such as maternal nutritional status are missing.

Provide the name of the country, instead of ‘western African”

Authors’ response:

Provide the name of the country, instead of ‘western African”

We amended the title as suggested:

Adverse birth outcomes and associated factors among newborns delivered in Sao Tome & Principe ~~a western African country~~: a case‒control study

Some other important risk factors such as maternal nutritional status are missing.

Some risk factors were not possible to collect since they are not included in the WHO´s ANC package for settings such as Sao Tome & Principe. Therefore, maternal factors such as gestational weight gain, BMI, and height were not feasible to include. During the face-to-face interviews I asked the participants if they Knew how many kg they might have gained during the pregnancy or their height, but participants did not know their height or weight. The main reason is that in Sao Tome & Principe, it is not frequent to find scales and stadiometers in health care facilities.

This is disclosed in the “Strengths and Limitations" section.

Please clarify the exposure variables. Some of the variables are mediators and it is unclear why they have been included in the analysis. The model building strategy is not clear.

The exposure variables (sociodemographic characteristics, preconception factors, ANC, intrapartum complications, health facility-related factors) for our case-control study were selected according to the literature, clinical practice and similar studies conducted in resource-constrained settings. For example,

i) Adane, et al. Adverse birth outcomes among deliveries in Gonder university hospital, Northwest Ethiopia. BMC Pregnancy Childbirth. 2014;14:90. http://www.biomedcentral.com/1471-2393/14/90

ii) Hailemichael HT, Debelew GT, Alema H, et al. Determinants of adverse birth outcome in Tigrai region, North Ethiopia: Hospital-based case-control study. BMC Pediatrics. 2020;20, 10. <https://doi.org/10.1186/s12887-019-1835-6>

iii) KC A, Basel PL, Singh S. Low birth weight and its associated risk factors: Health facility-based case-control study. PloS one. 2020;15(6):e0234907. <https://doi.org/10.1371/journal.pone.0234907>

Newborns’ characteristics and complications were described as we wanted to characterize our study population for these variables. These variables may be considered potential mediators, which means they are part of the causal pathway between the exposure variable and the outcome variable. The decision to include potential mediators in the analysis was due to the type of research question for this case-control study.

However, none of these potential mediator variables were included in the multivariable model. A note at the end of Table 3 adds to clarify this.

By including potential mediators, researchers can better understand the underlying mechanisms or pathways that are involved in the relationship between the exposure variable and the outcome variable. This helped to identify potential targets for interventions.

**Reviewers' comments:**

Reviewer's Responses to Questions

Comments to the Author

**1. Is the manuscript technically sound, and do the data support the conclusions?**

The manuscript must describe a technically sound piece of scientific research with data that supports the conclusions. Experiments must have been conducted rigorously, with appropriate controls, replication, and sample sizes. The conclusions must be drawn appropriately based on the data presented.

Reviewer #1: Partly

Reviewer #2: Yes

**2. Has the statistical analysis been performed appropriately and rigorously?**

Reviewer #1: Yes

Reviewer #2: Yes

**3. Have the authors made all data underlying the findings in their manuscript fully available?**

The PLOS Data policy requires authors to make all data underlying the findings described in their manuscript fully available without restriction, with rare exception (please refer to the Data Availability Statement in the manuscript PDF file). The data should be provided as part of the manuscript or its supporting information, or deposited to a public repository. For example, in addition to summary statistics, the data points behind means, medians and variance measures should be available. If there are restrictions on publicly sharing data—e.g. participant privacy or use of data from a third party—those must be specified.

Reviewer #1: No

Reviewer #2: No

Authors’ response:

The datasets used and analyzed during the current study are attached as “supporting information”.

**4. Is the manuscript presented in an intelligible fashion and written in standard English?**

PLOS ONE does not copyedit accepted manuscripts, so the language in submitted articles must be clear, correct, and unambiguous. Any typographical or grammatical errors should be corrected at revision, so please note any specific errors here.

Reviewer #1: No

Reviewer #2: No

Authors’ response:

We now submit a revised manuscript with American style.

Issues of grammar and wrong spelling were all rectified.

An additional effort was made with the help of an English-speaking health professional who assisted in improving the language of the paper now submitted.

We also submitted the manuscript to the AJE Academic Services of American Journal Experts.

We believe that with the changes made, the manuscript is now improved substantively.

**5. Review Comments to the Author**

**Reviewer #1:**

I commend the authors for their effort on such an important study in STP. The method and findings and just. However, more work needs to be done on the style and presentation in English to meet journals expectations. There is a need to be succinct and use less repetition. Some sentences and paragraphs are too chunky. I have made individual comments on the attached word document.

Authors’ response:

We truly appreciate Reviewer 1´s valuable contributions and feedback.

We now submit a revised manuscript with improved language that is much more succinct and without repetition. We were also consistent with the use of terms and their abbreviations throughout the text.

We amended the manuscript according to all Reviewer 1´s suggestions.

We believe that this paper will be very important for Sao Tome & Principe and similar countries.

Reply to Reviewer 1´s suggestions:

**Abstract** was amended as Reviewer 1 suggested: lines 31 and 33.

**Introduction** was amended as reviewer 1 suggested: line 169 from material and methods were incorporated in the introduction and were also reworded.

**Materials and methods** were amended as Reviewer 1 suggested: line 157, 158, 169

All material and methods sections were summarized and compressed as suggested. Repetitions were deleted.

**Results** were amended as Reviewer 1 suggested: we deleted repetitions. Prevalence was deleted.

Table 2 - for ANC characteristics was unformatted. The point estimate can now be properly seen. The text presented in the Results section was summarized and repetitions were deleted.

**Discussion** was amended as Reviewer 1 suggested: abbreviations were used instead of the terms. Misclassification of outcomes related to prematurity was further elaborated. Authors and references were cited accordingly.

Regarding the comment “What you mean by authors? Do you meant to say clinicians?”

*“Some authors even propose a cesarean section when there is a thick meconium-stained amniotic fluid to ensure a better outcome for the neonate even in the presence of normal fetal heart rate tracings on cardiotocography [55].”* *It is recommended in the following article:* 55.	Desai D, Maitra N, Patel P. Fetal heart rate patterns in patients with thick meconium staining of amniotic fluid and its association with perinatal outcome. Int J Reprod Contracept Obstet Gynecol. 2017;6(3):1030–5”

We decided to delete this information since this C-section indications are not always feasible in countries such as STP that have only one anesthesiologist.

**References:** All references were renumbered according to the changes in their new order of citation in the manuscript.

**Reviewer #2:**

Authors’ response:

We also appreciate Reviewer 2´s precious contributions and feedback.

1. Specify the study country, where it applies.

Authors’ response:

Done.

2. The entire draft needs to be rewritten and/or edited for more clarity for the flow and content of respective section.

Authors’ response:

Done. We now submit a revised manuscript with American style and issues of grammar and wrong spelling were all rectified.

An additional effort was made with the help of an English-speaking health professional who assisted in improving the language of the paper now submitted. We also submitted the manuscript to the AJE Academic Services of the American Journal Experts. We believe that with the changes made the manuscript is now improved substantively.

3. Reconsider the measurement of ANC as 8+ ANC is the latest development and its implementation may not be widely adopted, especially for births that happened around tail end of your data collection period.

Authors’ response:

We appreciate Reviewer 2´s comment.

We analyzed our study participants by ANC measurement of [1-4], [4-7] and [8+] ANC contacts.

We used these measurements since they translate the country´s reality, as we have previously found, in another study that we conducted that: “Antenatal care coverage in Sao Tome & Principe is a success compared to other SSA countries [8, 44]. Attendances are extremely high for a low-resource country. The minimum of eight ANC contacts, endorsed by the 2016 WHO´s guideline, was reached in almost forty percent. Moreover, 85.1% of the participants had four or more contacts when the rates reported in SSA for 4 visits were approximately 62% [44].”

Vasconcelos A, Sousa S, Bandeira N, Alves M, Papoila AL, Pereira F, Machado MC. Antenatal screenings and maternal diagnosis among pregnant women in Sao Tome & Principe-Missed opportunities to improve neonatal health: A hospital-based study. PLOS Global Public Health. 2022;2.

Available from: <https://doi.org/10.1371/journal.pgph.0001444>

4. Could it be possible to enhance the internal validity of the findings by incorporating additional 'distal' factors, such as SES and environmental factors, into the analysis? This could provide valuable contributions to the field as the current associations have already been established in OBGYN textbooks. Moreover, testing hypotheses on these distal factors may reveal some ‘unique and original’ insights for the field under consideration.

Authors’ response:

We were able to include all main sociodemographic variables recognized as being associated with ABOs (maternal age, education, occupation, marital status, baby´s father´s education, residence) and some environmental factors such as access to improved water and type of sanitation. During the mothers’ face-to-face interviews, we also collected other distal factors, such as the type of cooking fuel mothers used. This has been associated with ABOs, as we described in our manuscript Introduction: *“Lack of adequate ANC, household air pollution from unclean cooking fuels, open defecation, no access to improved water, violence, and other socioeconomic disparities are also considered important risk factors for ABO in sub-Saharan African (SSA) countries [15-17].”*

However, this factor of *“household air pollution from unclean cooking fuels”* was not included in the study, as most women (~80%) in the country use charcoal/coal as a cooking fuel, making comparison not feasible.

The SES variable analysis is depicted in the Results section and disclosed in the Discussion:

*“Studies differ in their observations on risk, and contrary to expectations, no association was found with the sociodemographic maternal characteristics, and there was no relationship between ABO and the maternal sanitation behavior and type of access to water, as reported in other LMICs studies [15, 17, 67].”*
